# Corroboration of a Major Role for Herpes Simplex Virus Type 1 in Alzheimer’s Disease

**DOI:** 10.3389/fnagi.2018.00324

**Published:** 2018-10-19

**Authors:** Ruth F. Itzhaki

**Affiliations:** Nuffield Department of Clinical Neurosciences, University of Oxford, Oxford, United Kingdom

**Keywords:** Alzheimer’s disease, senile dementia, herpes simplex virus, varicella zoster virus, population epidemiology, anti-herpes antiviral, fibromyalgia, epilepsy

## Abstract

Strong evidence has emerged recently for the concept that herpes simplex virus type 1 (HSV1) is a major risk for Alzheimer’s disease (AD). This concept proposes that latent HSV1 in brain of carriers of the type 4 allele of the apolipoprotein E gene (APOE-ε4) is reactivated intermittently by events such as immunosuppression, peripheral infection, and inflammation, the consequent damage accumulating, and culminating eventually in the development of AD. Population data to investigate this epidemiologically, e.g., to find if subjects treated with antivirals might be protected from developing dementia—are available in Taiwan, from the National Health Insurance Research Database, in which 99.9% of the population has been enrolled. This is being extensively mined for information on microbial infections and disease. Three publications have now appeared describing data on the development of senile dementia (SD), and the treatment of those with marked overt signs of disease caused by varicella zoster virus (VZV), or by HSV. The striking results show that the risk of SD is much greater in those who are HSV-seropositive than in seronegative subjects, and that antiviral treatment causes a dramatic decrease in number of subjects who later develop SD. It should be stressed that these results apply only to those with severe cases of HSV1 or VZV infection, but when considered with the over 150 publications that strongly support an HSV1 role in AD, they greatly justify usage of antiherpes antivirals to treat AD. Three other studies are described which directly relate to HSV1 and AD: they deal respectively with lysosomal changes in HSV1-infected cell cultures, with evidence for a role of human herpes virus type 6 and 7 (HHV6 and HHV7) in AD, and viral effects on host gene expression, and with the antiviral characteristics of beta amyloid (Aβ). Three indirectly relevant studies deal respectively with schizophrenia, relating to antiviral treatment to target HSV1, with the likelihood that HSV1 is a cause of fibromyalgia (FM), and with FM being associated with later development of SD. Studies on the link between epilepsy, AD and herpes simplex encephalitis (HSE) are described also, as are the possible roles of APOE-ε4, HHV6 and HSV1 in epilepsy.

## Introduction

The viral concept of Alzheimer’s disease (AD) proposes that herpes simplex virus type 1 (HSV1) in brain of apolipoprotein E gene (APOE-ε4) carriers accounts for some 60% of cases (Itzhaki et al., 1997). Most of the population is infected with this virus by the age of 70. The concept postulates that HSV1 travels to the brain probably in middle age, where it remains in a latent state, with very limited transcription and probably very low or zero protein synthesis. Reactivation from latency occurs intermittently, caused by events such as immunosuppression, peripheral infection and inflammation. Accumulation of the consequent damage—direct viral action and major inflammatory effects—leads eventually to the development of AD (Wozniak and Itzhaki, 2010).

The main initial discovery on which this concept was based was that HSV1 DNA was detectable in brain of both AD patients and elderly normal people (i.e., the latter were infected but were asymptomatic; Jamieson et al., 1991), the two groups differing in that most of the AD patients were APOE-ε4 carriers (Itzhaki et al., 1997). It was therefore suggested that APOE-ε4 carriers suffer either greater viral damage on reactivation or have poorer repair of such damage. In a striking parallelism in the PNS, APOE-ε4 was found to be a risk for cold sores (herpes labialis), which are caused mainly by HSV1 (Itzhaki et al., 1997). Also in genital herpes, caused usually by HSV2, APOE-ε4 is a risk for recurrence of genital ulcers (Jayasuriya et al., 2008). The subsequent finding that antibodies to HSV (these are known to be long-lived after herpes simplex encephalitis (HSE)) were present in cerebrospinal fluid from AD patients and age-matched controls showed that productive HSV1 infection had occurred, indicating that HSV1 is not a passive resident in the central nervous system (CNS; Wozniak et al., 2005). The data cannot be explained by a greater susceptibility of AD sufferers, or of APOE-ε4 carriers, to HSV1 infection, as the virus was present in brain at almost the same frequency in AD patients as in the controls, and was far more frequent among non-APOE-ε4 carriers than among APOE-ε4 carriers in the controls (although admittedly, the numbers in each category were very small).

Links between HSV1 action and AD (Tables 1, 2) include the discovery that the viral DNA is located very specifically within AD plaques (Wozniak et al., 2009a), and that the main component of plaques, beta amyloid (Aβ), accumulates in HSV1-infected cell cultures (Wozniak et al., 2007; De Chiara et al., 2010; Santana et al., 2012), and in the brains of HSV1-infected mice (Wozniak et al., 2007); subsequently others confirmed and extended these results (see review, Wozniak and Itzhaki, 2010). Taken together, the data suggest that HSV1 is a cause of Aβ products and plaques. We and others have shown too that the main component of tangles—an abnormal form of the protein called tau (P-tau)—accumulates in HSV1-infected cell cultures (Zambrano et al., 2008; Wozniak et al., 2009b; Alvarez et al., 2012).

**Table 1:** Main data on herpes simplex virus type 1 (HSV1) and AD from the author’s laboratory between 1991 and 2015.

Discovery	Reference
HSV1 DNA detected (by PCR) in brains of elderly controls and AD patients.	Jamieson et al. (1991)
HSV1 in brain of APOE-ε4 carriers confers high risk of AD. APOE-e4 is a risk for cold sores. First of several articles showing that APOE genotype modulates extent of microbial damage.	Itzhaki et al. (1997)
HHV6 DNA is present in AD brains.	Lin et al. (2002)
Intrathecal antibodies to HSV1 found in the elderly, showing that productive infection of HSV1 in brain has occurred.	Wozniak et al. (2005)
Aβ accumulation occurs in HSV1-infected cell cultures.	Wozniak et al. (2007)
HSV1 DNA is located specifically in amyloid plaques of AD brains.	Wozniak et al. (2009b)
AD-like tau (P-tau) accumulation occurs in HSV1-infected cell cultures.	Wozniak et al. (2009a)
HSV1 activates BACE1 via activation of PKR, then phosphorylation of eIF2-α.	Ill-Raga et al. (2011)
Acyclovir and other HSV1 replication-inhibitors reduce greatly the levels of Aβ and P-tau in HSV1-infected cell cultures.	Wozniak et al. (2011)
IVIG reduces greatly the levels of Aβ and P-tau in HSV1-infected cell cultures.	Wozniak and Itzhaki (2013)
Helicase primase inhibitor reduces greatly the levels of Aβ and P-tau in HSV1-infected cell cultures.	Wozniak et al. (2013)
Fucan reduces greatly the levels of Aβ and P-tau in HSV1-infected cell cultures.	Wozniak et al. (2015)
Interpretation of Taiwan population epidemiological data on HSV and risk of AD and antiherpes effects on development of senile dementia.	Itzhaki and Lathe (2018)

**Table 2:** Some major discoveries relating to HSV1 and AD between 2005 and 2018.

Discovery	Reference
Association of cognitive impairment with HSV1-seropositive APOE-ε4 in aged cardiovascular patients.	Strandberg et al. (2005)
HSV1 load/expression is greater in APOE-ε4 transgenic mice.	Burgos et al. (2006), Miller and Federoff (2008) Bhattacharjee et al. (2008)
Presence/levels of serum anti-HSV1 antibodies is associated with AD.	Letenneur et al. (2008) and Lövheim et al. (2015)
HSV1-infected cell cultures produce hyper-phosphorylated tau.	Zambrano et al. (2008)
Genetic links between HSV1 and host cells from GWAS.	Licastro et al. (2011), Carter (2013)
Aβ inhibits HSV1 DNA replication in cultured neuronal cells.	Bourgade et al. (2015)
HSV1 causes synaptic dysfunction if cultured cortical neurons.	Piacentini et al. (2015)
Lysosomal load increases and lysosomal function inpaired in HSV1-infected cell cultures.	Kristen et al. (2018)
HSV1-infection confers a risk of senile dementia and antiherpes antivirals strongly protect against SD.	Tzeng et al. (2018a)
High levels of HHV6 and 7 in AD brains HSV1& also HSV1, and they cause changes in several transcriptional regulators.	Readhead et al. (2018)
Aβ fibrillization occurs when Aβ oligomer enfolds HSV1 as a protective measure.	Eimer et al. (2018)

It should perhaps be stressed that the viral concept does not preclude a major role for Aβ and P-tau in the etiology of AD, even though their effects are still little understood; instead it suggests a cause of their accumulation—namely, HSV1 infection. Further, in HSV1- infected cells in culture, treatment with various types of antiviral have been found to decrease the level of Aβ and particularly, that of P-tau (see e.g., Wozniak et al., 2011). Usage of antivirals such as acyclovir (ACV), which inhibits viral DNA replication, showed that P-tau formation depends on viral DNA replication, whereas Aβ formation does not do so; inhibition of the latter by such agents probably occurs via inhibition of virus spread.

## Detection of HSV1 in Brain and Evidence for its Role in AD

The presence of HSV1 in brain is central to these concepts. Following its discovery in elderly brains by the author’s group, studies by five other groups confirmed its presence there (see review, Wozniak and Itzhaki, 2010). Other data too have provided confirmation—sometimes indirect, from very diverse types of approach (Table 2 and see review, Itzhaki, 2014), including studies on HSV1-infected APOE-transgenic mice or APOE-transfected cell cultures, GWAS, epidemiological investigations on anti-HSV1 IgG and IgM antibodies in serum from AD patients, or on infectious burden, and measurement of IgG avidity index (Agostini et al., 2016) as an indicator of reactivation (IgG presence indicates infection with HSV1, and IgM indicates HSV1 recent reactivation). The results showed an association between systemic infections and cognitive decline, with HSV1 particularly implicated, and many authors explicitly stating that their results supported a viral role in AD.

However, two recent articles (Olsson et al., 2016; Pisa et al., 2017) maintained that HSV1 is present in only a small proportion of brains of elderly people and AD patients. In the former study, the reason was probably usage of old fixed material, long duration of storage—known to be detrimental to PCR. However, in neither study did the authors specify the sensitivity of their PCR, so the level in some of their brain samples might well have been below their detection limit. The main topic of the other study was a search for fungi in brain; the authors stated that they detected HSV1 DNA in only 1 out of 10 brain samples (Pisa et al., 2017). However, as in the Olsson et al. (2016) study, the authors did not state the sensitivity of detection, and no recovery experiments were described, i.e., addition of HSV1 DNA to samples that were apparently virus DNA-negative to find if any contaminant was interfering with detection of the viral DNA. The second study sought also specific HSV1 proteins by immunohistochemistry (IHC), using fixed brain slices, and HSV1-infected HeLa cell cultures as “controls.” However, the level of virus and viral proteins in human brain would have been vastly less than in the infected cell cultures—so unsurprisingly, their IHC results were negative.

Another aspect linking HSV1 to AD, and relating also to the degradation of Aβ, is lysosomal impairment, which many studies have shown contributes to neurodegeneration, neurons being particularly susceptible to lysosomal damage. Very recently, Kristen et al. (2018) found that in cell cultures, HSV1 infection and also oxidative stress (OS) increased lysosomal load and impaired lysosomal function, the impairment including a reduced activity of lysosomal hydrolases and cathepsins, and in the case of OS, effects on the maturation of the cathepsins. Such changes could account for the accumulation of lysosomes and decreased functionality of lysosomal proteins, which are known to occur early in the development of AD. The authors pointed out that several polymorphisms associated with AD, such as APOE, ABCA7, CD2AP and Phosphatidylinositol Binding Clathrin Assembly Protein (PICALM) are associated also with the HSV1 life cycle, and that some of these lead to abnormalities in autophagy. All these data support the involvement of lysosomal damage in the development of AD, resulting in inefficient removal of toxic substances from cells, and they support the role of HSV1 in AD. The fact that the concentration of lysosomal proteins is known to be higher in AD patients’ brains and CSF might reflect attempts by cells to rectify the impairment of the lysosomal system.

Two other very recent publications which are consistent with the viral concept of AD have elicited much interest and publicity, resulting in some previously sceptical opponents of the viral concept conceding some possibility of its validity. The first, by Readhead et al. (2018), analyzed the transcriptomes of brain samples from AD patients and controls, using four independent cohorts from different geographical regions of the USA. They found that herpesviruses 6A and 7, and also HSV1, were present in elderly and AD brains, the levels of HHV6 and HHV7 being significantly higher in the AD samples than in the controls, in three of the four cohorts. Their results substantiate and augment earlier studies detecting HHV6 (Lin et al., 2002) and HSV1 (Jamieson et al., 1991) in elderly brains (which revealed a similar frequency of HSV1 in brain of AD patients and controls but a much higher HHV6 frequency in patients (see also review, Hogestyn et al., 2018). Readhead et al. (2018) found also an association of virus levels with clinical dementia rating, neurofibrillary tangle density and amyloid plaque density. Plaque size too was affected by virus presence, as they showed by suppressing the gene for miR-155, a neuroprotective micro RNA: miR-155 knockout mice were crossed with APP/PS1 mice and it was found that the progeny had more and larger plaques than the APP/PS1 controls. Importantly, their analysis of protein and mRNA levels suggested that infection with these viruses causes changes in several transcriptional regulators (including several regulators of APP processing and AD risk-associated genes such as gamma-secretase subunit presenilin-1 (PSEN1), BACE1, Clusterin (CLU), PICALM. These data too are consistent with earlier studies using GWAS on associations between microbes, particularly herpesviruses, and AD, as described by Licastro et al. (2011) and Carter (2013). Lin et al. (2002) raised the possibility that HHV6 infection might be merely an opportunistic infection, but suggested that it was more likely that HHV6 acts in concert with HSV1, as studies by others had shown that HHV6 augments the damage caused by other viruses in animal tissue and in cell cultures. Also, the data of Readhead et al. (2018) argue against HHV6 and HHV7 being merely opportunistic infections, in that they reveal association between virus levels and levels of various characteristic AD features, as mentioned above.

The second article, by Eimer et al. (2018), extends greatly previous antimicrobial research by the group, and by Bourgade et al. (2015) specifically on the antiviral properties of Aβ, the deposition of which they describe as an innate immune response to infection. The authors found that HSV1 and HHV6 can induce amyloid plaque production in infected mice within 24–48 h, and that Aβ oligomers inhibit HSV1 infection in 3-D human neural cell culture models, and protect 5XFAD transgenic mice from acute viral encephalitis. They showed also that Abeta, probably in the form of a soluble oligomer, ensnares the virus through its heparin-binding domain via the viral envelope glycoproteins, thereby protecting brain cells from infection; fibrilization of the amyloid cloak occurs rapidly. This cloaking echoes the suggestion by Robinson and Bishop (2002) that amyloid acts protectively by entombing pathogens—a then heretical notion that was either ignored or derided. Cloaking is consistent also with the finding that in AD brains, HSV1 DNA is very specifically located within amyloid plaques (Wozniak et al., 2009a).

Both sets of authors acknowledge that their very interesting data show *association* of herpesvirus with AD but cannot prove *causality*. In contrast, and very importantly from the viewpoint of AD patients especially, population studies in Taiwan published in the last 12 months *do* provide evidence of causality.

## Evidence From Population Epidemiological Studies for a Role of HSV1 and Other Herpes Viruses in Dementia

Three very significant publications have appeared in the current year, all providing data on the health and illnesses of a population over several years—information which is not available in the UK^1^ nor, probably, in most other countries. In Taiwan, there are records of over 99% of the population and it seems that the data are being exhaustively mined by Taiwanese epidemiologists for links between, for example, various viruses, and certain chronic disorders, including senile dementia (SD). These are yielding important results. All three articles describe data on herpes virus infections—a family of viruses that affects the vast majority of people worldwide, at least by the age of 60 or so. These viruses, once in the body, are harbored there for life, usually in a latent state but can be reactivated to an active, replicative state. Only a certain proportion of those infected actually show overt symptoms; the remainder are asymptomatic (as is the case for many or perhaps all microbial diseases). In the Taiwan publications, the word “infection” is used to denote people who showed overt signs of the disease such as shingles or recurrent cold sores or genital sores, rather than for all those who carry the virus asymptomatically in either a latent or an active, productive state. Also, the term “SD” is used rather than AD because in some cases the diagnosis was uncertain.

Two of the articles investigated varicella zoster virus (VZV) infection in relation to long-term neurocognitive changes and the development of dementia. VZV causes chicken pox, but after acute infection it remains in the body lifelong in latent form, and in some people in older age it reactivates, causing shingles, which both sets of authors referred to as herpes zoster (HZ) and the virus as HZV. The first article, by Tsai et al. (2017), investigated 846 patients (mean age 62.2 years) who were diagnosed with HZ ophthalmicus (HZO) in 2005 and who developed dementia in the following 5 years. The development of dementia was compared with that of an age-matched control group of 2,538 subjects during the same 5-year period. The percentage of patients with HZO who developed SD was 4.16%, whereas that of the controls was only 1.65% (*P* < 0.001), and the crude hazard ratio of developing SD within 5 years of HZO diagnosis was calculated to be 2.97 after adjustment for patients’ characteristics and co-morbidities. This represents a remarkably high risk of developing dementia amongst HZO sufferers.

In the second article, by Chen et al. (2018), 39,205 patients with HZV, age range 54–90, were diagnosed during the period 1997–2013 and were followed over an average period of 6.2 years. The incidence of dementia was compared with that of 39,205 controls (mean age of both groups was 63.5 years). The hazard ratio was only very small, namely, 1.11. A possible explanation for this marked difference from the HZO results is that in HZO the virus is more likely to enter the brain and cause damage there than in HZV infection. However, HZ patients who were treated with antiherpes antivirals—acyclovir, valacyclovir, tromantadine, famciclovir—showed a dramatic decrease in incidence of dementia to about a half of that in the untreated group, adjusted HR, 0.55; 95% CI, 0.40–0.77, (*P* < 0.0001).

The third and most striking article, and the one directly relevant to HSV1 and AD was by Tzeng et al. (2018a). The authors investigated 8,362 subjects aged 50 years or over during the year 2000 who were newly diagnosed with HSV1 or HSV2 infections—presumably recurrent herpes labialis or genital ulceration, on at least three outpatient visits within the year. The control group of 25,086 age- and gender-matched subjects had no HSV infection during the year 2000. The incidence of dementia in both groups was investigated during the 10 years 2001–2010. The risk of developing SD in the HSV group was found to be 2.56-fold greater, 95% CI 2.351–2.795; *P* < 0.001), similar to the risk associated with ophthalmic HZO infection. The main effect was seen in those with HSV1 rather than HSV2 infections. Subtypes AD and vascular dementia had similar risk profiles.

Even more strikingly, a group of HSV-infected patients (*N* = 7, 215) who had been treated with one of various anti-herpes agents (acyclovir, famciclovir, ganciclovir, idoxuridine, penciclovir, tromantadine, valaciclovir (VCV—the biodrug of ACV, which is better absorbed) and valganciclovir), showed a dramatic reduction of almost 10 fold in the later incidence of SD compared with those who received no treatment (*N* = 1,147; relative risk factor = 0.092, 95% CI 0.079–0.108, *P* < 0.001). In the subgroup with antiherpetic medications, 419 (5.80%) developed dementia in the longitudinal follow-up within 10 years. In the subgroup without antiherpetic medication treatment, 325 (28.33%) developed dementia in the same follow-up period; relative risk factor = 0.092, 95% CI 0.079–0.108 (*P* < 0.001).

Thus, antiherpetic medications, either over-all (adjusted HR: 0.092, 0.079–0.108, *p* < 0.001) or individual antivirals, were associated with decreased risk of developing dementia (Table 3). The protection was greater in those treated for longer time periods (over 30 days vs. less than 30 days) but the effect is remarkable anyway in showing that treatment for periods of relatively short duration could prevent the (presumably) later processes in brain which ultimately led to the development of AD. In the case of HZ patients, whether the antiviral treatment acted directly against HZV action if in brain, or against HSV1 reactivated in brain by HZ-induced inflammation is unknown; the latter seems more likely in view of the fact that HZV DNA has not been detected in any elderly or AD brains (Lin et al., 1997). In theory (though probably not in practice), a direct effect of any microbe in brain as opposed to an effect of HSV1 reactivated by microbe-induced inflammation could be tested by treatment with an antiviral that targets only HSV1, but current antiherpetics are not HSV1-specific, and anyway, such treatment might be ethically dubious.

**Table 3:** Relative risks for development of senile dementia in herpes zoster and HSV cases, and after anti-viral treatment.

Type of illness/infection	Relative risk
**Herpes zoster ophthalmicus**
Developing SD within 5 years of HZO diagnosis vs. age-matched controls.	2.97
**Herpes zoster**
Developing SD within 6 years of HZ diagnosis vs. age-matched controls.	1.1
Developing SD in AVT-treated HZ patients vs. untreated HZ patients	0.55
Herpes simplex types 1 and 2*
Developing SD within 10 years of HSV diagnosis vs. HSV-negative subjects.	2.564
Developing SD in AVT-treated HSV patients vs. untreated HSV patients.	0.092

*All severe cases.

The mechanism of this action is unknown. To speculate, it might involve prevention by anti-viral treatment (AVT) of the virus reaching the CNS—based on the assumption that this passage occurs in middle age, when the immune system starts to decline. This seems plausible as all the subjects with HSV1 infection were ≥50 years old and were selected on the basis of having had ***newly diagnosed*** HSV1 infection during the period January 1, 2000 to December 31, 2000. The virus might not therefore have reached their brain, as although primary infection must have occurred before the diagnosis—and possibly much earlier (for in some cases overt symptoms occur well after primary infection), the virus level might have been too low to lead to passage to the brain. AVT, which stops HSV1 replication, would have reduced the level in the periphery, thereby decreasing the chance of its reaching the brain. However, it seems likely that AVT, rather than blocking viral passage, probably delayed it. This could be checked by extending the survey further, perhaps from 2010 to 2017, to find if the number of dementia cases increased (though there would be an increase also in death rate with age). Investigations *post mortem* to seek HSV1 DNA in the brain of any such subsequent cases of dementia, and in some of those who remained free of the disease, might help to clarify the effect of AVT.

All these data, together with the data on HSV1 presence in a high proportion of elderly brains (Jamieson et al., 1991), and its association with APOE-ε4 in AD patients (Itzhaki et al., 1997), strongly support a causal role of HSV1 in AD, and support also the likelihood that antiherpetic treatment—probably more effective if combined with anti-inflammatory treatment—could be used to prevent disease occurrence or to slow disease progression. However, there are no data on the effect of antivirals on those already suffering the disease. Indeed, the fact that antiviral treatment was very effective in reducing the incidence of dementia, when given before any overt signs of dementia were apparent, suggests that treatment to prevent the disease would be more likely to succeed if carried out before middle age (say between ~30–40 years), even if the treatment were for only a relatively short period, rather than if given after the onset of AD. In the UK, the proportion of 30–40 year old group who are HSV1-seropositive is at most ~70% (Looker et al., 2015), and the proportion of that age-group who are carriers of an APOE-ε4 allele is ~25%, so overall, only approximately 18% (0.7 × 25%) of the age group would be most at risk and therefore most likely to benefit from antiviral treatment, which, it should be noted, is very safe and relatively inexpensive.

Another uncertainty exists because the treated group comprised only those who had severe herpes labialis or severe genital ulcers (as they were selected only if they had made at least three outpatient visits in the year 2000). What proportion these severe cases constituted of those who eventually developed dementia and were HSV1-seropositive and APOE-ε4 carriers, is uncertain, though it is probably very low. Thus, it is unknown if subjects who are HSV1-seropositive and APOE-ε4 carriers, but who are only mildly affected or are asymptomatic, would be as susceptible to treatment. Nonetheless, it seems extremely likely that the results of the Taiwanese studies would apply also to the many AD patients who, although HSV1-seropositve, never previously displayed overt symptoms of the infection.

Despite the uncertainties mentioned above, as well as others such as what future modes and timing of treatment should be used, these epidemiological results represent a very important new step to the problem of understanding and treating those AD cases probably caused by HSV1 (Itzhaki and Lathe, 2018). It is worth stressing though that these data and the preceding evidence for a role of HSV1 in AD, do not preclude a role for bacteria, in particular, *Borrelia, Chlamydia pneumoniae*, and some oral bacteria, which are probably the microbes most strongly implicated in AD (see review, Miklossy and McGeer, 2016): one or more such microbes might be involved, leading to the disease in the sizeable proportion of AD patients whose illness is *not* accounted for by HSV1 (in combination with APOE-ε4).

## Recent Data from Other Diseases Relevant to HSV1 and Cognitive Decline and to Anti-viral Treatment

Three other publications are particularly interesting, even though none deals directly with the issue of HSV1 in brain and AD. The first relates to certain cognitive features and HSV1. A number of studies have shown that HSV1-seropositivity is associated with cognitive dysfunction—particularly in schizophrenic (SZ) patients. Bhatia et al. (2017) investigated temporal changes in various cognitive features over a period of 1–3 years, mean follow-up time 1.93 years, and also the effect of VCV, comparing the changes in HSV1 seropositive and seronegative SZ and control subjects. Emotion Identification and Discrimination (EMOD), spatial memory and spatial ability were investigated in 131 HSV1-seropositive and 95 HSV1-seronegative people, mean ages 35 and 32, respectively. EMOD was defined as ability to discriminate between emotions and is considered an important component of social cognition (Gur et al., 2010).

SZ subjects had significantly lower scores in all cognitive domains. The HSV1-infected subjects had significantly lower scores than uninfected subjects for the above cognitive features, regardless of SZ diagnosis (*p* = 0.025, 0.029, 0.046, respectively, and their values for EMOD decreased significantly more rapidly (*p* = 0.033).

In the VCV study, 30 subjects were given VCV orally at 1.5 gm twice daily for 16 weeks, and 32 subjects were given placebo, while continuing with standard antipsychotic treatment. The results indicated improvement in EMOD among HSV1 infected persons with SZ, following VCV treatment (*p* = 0.048, Cohen’s *d* = 0.43). The authors concluded that HSV1 infection was associated with dysfunction in various cognitive features at study entry in SZ patients and controls, and with greater temporal decline in EMOD.

Another interesting study investigated fibromyalgia (FM) in relation to HSV1. FM is characterized by chronic widespread pain, fatigue, sleep disruption, and cognitive impairment. Pridgen et al. (2017) treated patients with the antiherpetic famciclovir (FCV), a nucleoside analog, in combination with a Cox-2 inhibitor, celecoxib (combination referred to as IMC-1), in a multicenter trial. Usage of the anti-herpes antiviral agent was based on the hypothesis that the disorder is caused by intermittent reactivation of latent HSV1 because of stress, etc. Celecoxib was used not only because of its direct Cox-2 inhibition but also because it has anti-herpes action. Several herpes viruses, including HSV-1, are known to upregulate COX-2 (Liu et al., 2014), and virally induced upregulation of COX enzymes is important for efficient HSV-1 replication. COX-2 inhibition reduces the severity of primary herpes virus lesions and inhibits reactivation of latent infections (Higaki et al., 2009). FM patients, mainly caucasian and female, age range 18–70 years, were enrolled in a 16-week, double-blinded, placebo-controlled, proof-of-concept trial held in 12 centers. Randomized patients received either IMC-1 or placebo. Fifty-seven patients completed the 16-week course of treatment with IMC-1 and 45 patients received a placebo (mean ages 51 and 48 years, respectively). The outcome was assessed by standard ratings of pain, fatigue and depression at baseline and weeks 6, 12 and 16 of the study.

The data showed a significant decrease in FM-related pain in IMC-1-treated patients compared with those given the placebo. The safety and tolerability profile for IMC-1 were encouraging. The authors commented that the two drugs when used in combination, might have acted additively and/or synergistically, thereby increasing the efficacy. Previous investigators using them separately had found that in contrast neither drug alone was efficacious in the treatment of FM. They concluded that their results supported the hypothesis that herpes virus infections may contribute to this syndrome.

The third article concerns FM and dementia, pursued by Taiwanese investigators again using the country’s insurance data, the rationale being that inflammation-related diseases or other pain disorders, such as headaches, have been shown to be associated with increased risk of dementia. Tzeng et al. (2018b) investigated 41,612 subjects with FM diagnosed between January 1, and December 31, 2000, and 124,836 controls without FM, matched for age, sex and index year. All subjects were aged 50 years or over. Patients were selected on the basis of having made at least three outpatient visits within the 1-year study period for FM or other co-morbidity. The risk of developing dementia during the 10-year follow-up period to December 31, 2010, was investigated. The results showed that FM was associated with increased risk of all types of dementia: 1,704 of the 41,612 FM patients (21.23 per 1,000 person-years) developed dementia, compared with 4,419 of the 124,836 controls (18.94 per 1,000 person-years). After adjustment for sex, age, etc. the hazard ratio was calculated to be 2.77 (95% CI: 2.61–2.95, *P* < 0.001). For the individual types of dementia, the risk for AD was 3.35-fold, for non-vascular dementia 3.14-fold (a group which, they acknowledge, might have included some wrongly diagnosed AD patients), and for vascular dementia 2.72-fold.

Tzeng et al. (2018b) described several possible limitations, and therefore concluded that the findings suggest association of FM with dementia, rather than causation, and that a longer follow-up period would help to clarify the long-term risks. However, in view of the study by Pridgen et al. (2017) suggesting that herpes virus infections may contribute to FM, the linking factor—and probable causative agent—is HSV1.

## HSV1 Infection of Mice

Although there is increasing evidence in humans that long term HSV latency correlates with increased risk of neurodegenerative disease, few animal studies have focused on correlating long term HSV infection in the CNS with functional cognitive/behavioral endpoints. Beers et al. (1995) published the first report providing evidence that spatial memory deficits were associated with HSV infection in Lewis rats that had recovered from encephalitis; this study was performed relatively soon after recovery from primary infection and long-term effects were not evaluated. An ongoing study is investigating whether long term-HSV latency results in measurable differences in cognitive performance in human-APOE-ε4 targeted knock-in transgenic mice (huApoE4; Sawtell et al., 2018). In two independent studies, two groups of mock infected and two groups of HSV-1 17syn + infected (via the ocular route) huApoE4 mice were utilized. Mice were monitored for overall health status, including weight, during the entire study. With the exception of minor blepharitis during acute stage of infection, no differences in overall health status between mock and HSV-1 infected groups were observed. Signs of encephalitis were not observed during acute infection and mortality from viral infection did not occur. HSV latency in the trigeminal ganglia and throughout the CNS was demonstrated at 40 days post infection by real-time qPCR. At 12 months post infection, groups of mice (*n* ≥ 16/group) were evaluated in a number of tests including the Morris Water Maize (MWM) which tests hippocampus-dependent spatial learning and memory. Striking and significant differences in both studies between HSV infected and mock infected groups were observed in the MWM, suggesting alteration in hippocampal function. Examination of hippocampal region revealed an ~8-fold increase in focal Aβ deposits. The investigators conclude that these behavioral studies draw a solid link between long term HSV latent infection and cognitive impairment in the context of the huApoE4 allele.

## Links Among Epilepsy and AD, APOE, HHV6, HSV1 and Herpes Simplex Encephalitis (HSE)

Increasing numbers of publications are linking epilepsy and AD in showing that seizure-like activity in the brain is associated with some of the cognitive decline seen in AD patients, and that seizures are more common in AD than in the general population. Also, the risk factors, APOE-ε4, HSV1 or HHV6 are increasingly implicated in both disorders. The three latter factors are discussed below.

AD patients have an increased risk of epilepsy and almost 50% have abnormal electrical activity in the brain, which does not cause a seizure but is detectable by brain scan technology. Lam et al. (2017) pointed out that amyloid plaques, characteristic of AD in their numbers in brain, were first described in 1892, in patients with epilepsy, and that AD and epilepsy both impair cognition and show overlapping patterns of cellular neurodegeneration and hypometabolism in the temporal lobe. They added that interneurons are among the first to die in AD mesial temporal cortex and that the ensuing degradation of synaptic connectivity and circuit remodeling could contribute to memory storage and retrieval. Intermittent temporal lobe dysrythmia could therefore account for early fluctuations in cognition in AD patients. The authors investigated mesial temporal activity in two AD patients with fluctuating cognition but with no previous history of seizures, using intracranial foramen ovale electrodes, and detected clinically silent hippocampal seizures and epileptiform spikes during sleep, a period when both were most likely to interfere with memory consolidation. They suggested that early development of occult hippocampal hyperexcitability might contribute to the pathogenesis of AD.

In a recent feasibility study (Musaeus et al., 2017), an anti-epileptic drug was tested for its potential impact on the brain activity of patients with mild AD in a double-blind within-subject study. Seven patients were investigated on three separate occasions: their baseline EEG was examined and then they were injected with either a placebo or with the anti-seizure drug levetiracetam, at either a low dose (2.5 mg/kg) or a higher dose (7.5 mg/kg). Each patient eventually got one dose of each type, in random order. After injection, patients underwent magnetic resonance imaging (MRI) to measure blood flow in the brain to quantify brain activity and detect its location in the brain, and they took standard cognitive tests for memory, executive functioning, naming, visuospatial ability and semantic function, all of which are affected in AD. The higher doses of the anti-seizure drug were found to normalize abnormalities in the patients’ EEG profiles, increasing brain wave frequencies that had been abnormally low, and decreasing those that had been abnormally high. Although the authors found no improvement in cognitive function after a single dose of medication, they plan a longer and larger study.

There is also an amyloid connection between epilepsy and AD: Joutsa et al. (2017) investigated 41 people who had suffered childhood-onset epilepsy (one in a hundred develop the disorder before age 18), and were then followed for 50 years until late middle age, and 46 matched population-based controls, using positron emission tomography scanning. The aim was to find if there was a predisposition to development of progressive neurodegenerative disorders such as AD, as indicated by Aβ accumulation, and to find if APOE genotype is a factor. The authors reported that the middle-aged adults who had developed childhood epilepsy had more amyloid plaques in their brains than matched controls without epilepsy. Plaque accumulation was especially great in APOE-ε4 carriers. The subjects had had a variety of different epilepsy syndromes and were in remission. Many had not had anti-epileptic drug therapy for decades which, the authors suggested, linked the increased brain Aβ to the pathophysiology of epilepsy rather than to seizure control or duration of active epilepsy, and might help to explain why childhood epilepsy could lead to cognitive disorders such as AD.

There have been a number of studies specifically on the involvement of APOE, and also of HSV1 and HHV6, in epilepsy. The role of APOE in epilepsy development is still controversial, some studies showing that ApoE-ε4 is associated with an increased risk of medically refractory epilepsy, of late post-traumatic seizures and of non-lesional mesial temporal lobe epilepsy (MTLE), while other studies found no association in non-lesional TLE patients nor in MTLE with hippocampal sclerosis (MTLE-HS) patients (Leal et al., 2017). An association between this isoform and age of onset of temporal lobe epilepsy was found in several studies; also, the APOE-ε4 allele has been associated with cognitive impairment in epileptic patients. Leal et al. (2017) aimed to elucidate the importance of febrile seizures (FS) and the role of APOE in MTLE-HS development. They described MTLE with hippocampal sclerosis (MTLE-HS) as the most frequent pharmaco-resistant epilepsy, with most HS patients having suffered CNS infection, head or birth trauma or FS, the latter being the most common injury. Their results showed no differences in APOE-ε4 frequencies between MTLE-HS patients and controls or between MTLE-HS subgroups, but APOE-ε4 carriers had an earlier MTLE-HS onset, as did MTLE-HS patients with FS antecedents compared with non-FS antecedents. They concluded that although APOE-ε4 and FS might not be involved in aetiopathogenic mechanisms of MTLE-HS, these factors could speed up disease development in predisposed individuals.

Other mainly negative findings included that of Li et al. (2016) who investigated Han Chinese and found no association between APOE-ε4 carriage and the age of onset, duration of epilepsy, frequency of seizure, febrile convulsion history, or hippocampal sclerosis, although they suggested that the ε4 allele was a possible risk factor for non-lesional MTLE. Also, Lavenex et al. (2016) detected no association between APOE polymorphisms and FS.

As to a viral involvement in epilepsy, Wipfler et al. (2018) carried out a meta analysis of eight publications on HHV6 and MTLE, all of which had used surgically removed tissue samples from pharmaco-resistant patients. HHV-6 DNA was detected in brain of 19.6% of all MTLE patients compared to 10.3% of all controls (*p* > 0.05). As the authors state, these data indicate an association between HHV-6 DNA and MTLE, although whether it involves HHV6 types A or B or both is unknown, as also is whether the association is causal.

HSE causes epilepsy and epilepsy surgery causes HSE recurrence. HSE is caused by HSV1 and is the most common type of viral encephalitis. It is an acute, rare but often fatal disease of the brain. In the last few decades, treatment of HSE by ACV and other antivirals has decreased mortality, but morbidity in survivors is still high. In the author’s previous review (Itzhaki, 2017), it was pointed out that HSE is a major cause of seizures, the occurrence of which is probably underestimated because of their frequent subtlety. Unprovoked seizures often occur in the post-acute phase (21 days from onset of initial symptoms) and they resist treatment. If seizures occur during the acute phase, there is a greater risk of post-encephalitic epilepsy, and hence of poor long- term prognosis (Sellner and Trinka, 2012).

In a reverse effect, surgery for treatment of epilepsy can cause the relapse of HSE, as described in several case reports: Bourgeois et al. (1999), Kim et al. (2013), Uda et al. (2013), Lo Presti et al. (2015), de Almeida et al. (2015) and Alonso-Vanegas et al. (2016). HSE relapse presumably occurs because of reactivation of existing HSV1 DNA in the brain caused by axonal cutting—a known reactivator of the virus.

HSE has some links with AD, in that it leads not only to seizures but also to memory deficits and behavioral changes, resembling some of the changes seen in AD patients. Thus, there are functional inks between HSE sequelae and AD, links between AD and epilepsy, and HSE and epilepsy, probable links between epilepsy and herpes viruses, possible links of epilepsy also with specific APOE alleles, and much evidence linking HSV1 to AD. The episodes of HSV1 reactivation which are postulated to occur in brain of the elderly must necessarily be very limited in extent as otherwise, they would lead to overt encephalitis. In view of these connections, it seemed reasonable to consider that HSE occurrence in APOE-ε4 carriers might lead to AD, although it would be rare because of the rarity of HSE (approx 1–3 cases per million population). Relevant literature was therefore searched to find if people who had suffered from HSE have a greater risk of developing age-related cognitive decline, and specifically of dementia or AD. Four published studies and one unpublished survey showed an increase in dementia or, specifically, of AD, amongst survivors of HSE, suggesting that the survivors might have shared another characteristic which added to a risk conferred by HSE—possibly, an APOE-ε4 allele, but unfortunately, none of the studies investigated the APOE genotype of their subjects (Itzhaki and Tabet, 2017).

There appear to be only two publications on APOE genotypes of HSE patients, one of which implicated APOE-ε2 as a risk (Lin et al., 2001). This would not necessarily invalidate the APOE-ε4 hypothesis, as AD might develop mainly in those HSE patients (some half of the total) who are not APOE-ε2 carriers. However, a second study found no significant difference between HSE patients’ genotypes and those of controls (Nicoll et al., 2001). The reason for the difference is unknown. As to dementia occurring also amongst survivors of non-HSE encephalitis (caused by other herpesviruses, bacteria or parasites), possibly this is a consequence of encephalitic damage in the CNS, which might well cause reactivation of latent HSV1 if present.

## Treatment of HSE With Acyclovir and Relevance to the Treatment of HSV1-Seropositive, APOE-ε4 AD Patients

The standard treatment for HSE is intravenous acyclovir, following trials in the 1980s which assessed its efficacy. It is strongly recommended that ACV should be given as soon as possible during an attack (or even before the diagnosis is certain, in suspected cases) because its usage leads to a striking drop in mortality. However, all too often there are serious sequelae of the illness, as mentioned above. As to the efficacy of longer-term treatment than the standard 14–21 days, a clinical trial of neonates with “HSV disease with CNS involvement” showed that prolonged oral dosing of ACV for 6 months after the usual 14–21 days greatly improved neurological outcomes (Kimberlin et al., 2011). However, another study found, in contrast, that valacyclovir treatment given for 90 days after standard intravenous ACV did not benefit adult HSE patients (Gnann et al., 2015; who were given 2 g thrice daily or placebo tablets). The primary endpoint was survival, with no or mild neuropsychological impairment at 12 months, as measured by the Mini-Mental State Examination (MMSE), and the Mattis Dementia Rating Scale (MDRS). To explain this unexpected result, Tyler (2015) pointed out that the patients in the study by Gnann et al. (2015) were a select group, described by him as a relatively high-functioning subset of HSE survivors. No seriously ill patients were enrolled, so whether such patients or those with associated immuno-compromising conditions might have benefitted is unknown. Nonetheless, the patients in the adult trial, treated and untreated, showed a remarkable extent of recovery. By 2 years post-illness, some 90% of the subjects had no or only mild impairment, as judged by either of the score systems. In fact, most of the improvement occurred within the first 90 days.

This high recovery group represents probably only a small minority: Gnann and Whitley (2017) have estimated that only 40%–55% of sufferers are able to resume activities of daily living at 12 months. To improve the high morbidity, they suggest the usage of combinations of ACV—or VCV plus immuno-modulatory drugs to reduce ongoing inflammation. A major possibility would be treatment with the combination IMC-1 used by Pridgen et al. (2017; see above).

As to the effects of treating AD patients long term, ACV causes few side-effects except in renally impaired patients; these should therefore be excluded from relevant trials. No ill effects were seen when VCV was used for a 2 year period at a dosage of 3 g per day, in a clinical trial designed to investigate its efficacy in treating multiple sclerosis (Friedman et al., 2005) and Pridgen et al. (2017) found the safety and tolerability of their IMC-1 in the 4-month treatment of their patients to be satisfactory.

## Conclusions

Further population epidemiological work would be invaluable for understanding the role of microbes, in particular HSV1, in AD. Using the Taiwan records, or those of any other country with comparable information, the subsequent development of dementia amongst subjects who had suffered mild herpes labialis or genital herpes could be investigated, although they would be far less likely to be documented, and therefore much less identifiable than severe cases. However, investigation of even asymptomatic HSV-seropositive people vs. HSV-seronegative people would be informative, although by the age of 60 the latter would comprise only a very small minority. Also, individuals could be selected who had suffered severe peripheral infections, on the basis that the inflammation thus caused could lead to inflammation in the brain, and reactivation of any latent microbe there. Of particular interest would be those who had suffered HSE, and also epilepsy patients—even those in whom no virus infection had been reported. If tissue, blood, or salve samples were available, APOE genotypes could be determined for any association with other characteristics.

Clearly, the types of antiviral which might be used for treating AD should be carefully chosen, especially if combined with an anti-inflammatory agent, as well as the duration of treatment and stage at which their usage would most effective. Even if the effects were merely a delay in onset of the disease, this would still be enormously beneficial for patients, carers and the economy. Of course, vaccination against HSV1 would be the better option, as prevention of disease is better than cure. Unfortunately, however, there is currently no vaccine for HSV1 and any vaccine trial would presumably have to extend for many years to find the outcome.

Research data on a microbial cause of AD have been ignored or dismissed for three decades, very unfortunately for those who developed AD during that period and who therefore had no chance of benefitting from the information. Surely, now is the time to rectify the situation by determining and then using the best means of treatment at hand.

## Author Contributions

The author confirms being the sole contributor of this work and has approved it for publication.

## Conflict of Interest Statement

The author declares that the research was conducted in the absence of any commercial or financial relationships that could be construed as a potential conflict of interest.

## Footnotes

^1^The present author sought relevant population information on HSV1, antiviral usage and AD in the UK in the 1990s but it was not available.
